# Synovial Chondromatosis of the Shoulder: Report of Two Cases

**DOI:** 10.1055/s-0044-1790596

**Published:** 2024-12-27

**Authors:** Daniel Ferreira Fernandes Vieira, Beatriz Begnini Borsato, Luís Felipe Baldinu Caramujo, Paula de Andrade Castello, João Paulo Fernandes Guerreiro

**Affiliations:** 1 Disciplina de Ortopedia, Pontifícia Universidade Católica do Paraná (PUC/PR), Londrina, PR, Brasil; 2 Hospital de Ortopedia Uniort.e, Londrina, PR, Brasil; 3 Escola de Medicina, Pontifícia Universidade Católica do Paraná (PUC/PR), Londrina, PR, Brasil

**Keywords:** arthroscopy, shoulder, synovial chondromatosis, synovial membrane

## Abstract

Synovial chondromatosis is an uncommon, progressive, benign condition favoring synovial metaplasia resulting from the production of cartilaginous tissue as loose bodies within the joints. In rare cases, it can affect the interior of the shoulder joint and present with pain, edema, and impaired mobility. The diagnosis is challenging, requiring imaging techniques. Its confirmation often occurs only after surgical treatment and anatomopathological examination. The authors report two cases of patients with similar clinical presentations of pain, edema, and mobility loss in the shoulder joint. The investigation included imaging exams, such as radiographs and magnetic resonance imaging, and an anatomopathological examination confirming the diagnostic hypothesis. Arthroscopic surgical treatment with synovectomy and removal of loose bodies followed by physical therapy rehabilitation resulted in clinical improvement in both patients. This report emphasizes the importance of investigating suspected synovial chondromatosis due to its nonspecific clinical presentation. Comparing our outcomes with the literature, we concluded that surgical treatment with synovectomy, loose body removal, and physical therapy is effective, and that long-term outpatient follow-up is necessary to detect recurrence.

## Introduction

Synovial chondromatosis (SC) is an exceptional arthropathy, usually monoarticular, resulting from the proliferation and metaplasia of synovial tissue and the formation of loose cartilaginous bodies in the tendon sheath or joint spaces. 1 2 3 The involvement pattern includes diarthrodial joints, especially the knee, hip, and elbow; descriptions of shoulder involvement are scarce in the literature. 2 SC etiology remains unknown, and the highest SC incidence occurs in the third to fifth decade of life. In addition, SC is three times more common in men. 3 The clinical picture consists of pain, crepitus, edema, and joint movement limitation, 2 often with no apparent cause. 1 Malignant transformation is uncommon, and there is no direct relationship with trauma or inflammatory processes. 4 Clinical diagnosis is difficult, as history and physical examination findings are not specific. Therefore, imaging methods, including radiography, computed tomography, and magnetic resonance imaging (MRI), become essential to identify the different types of SC lesions and stages. 5 Diagnostic confirmation relies on histological examination of the synovial tissue, and surgery is the treatment of choice for symptomatic patients. 1 3

## Case Reports

**Fig. 1:**
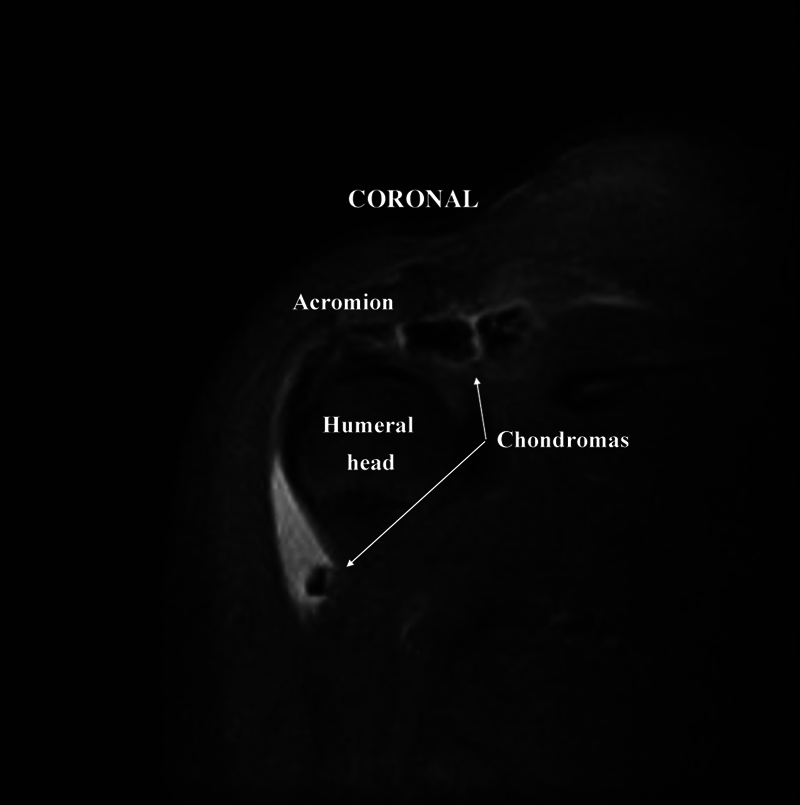
Magnetic resonance imaging of the shoulder (coronal view, T2-weighted image).

**Fig. 2:**
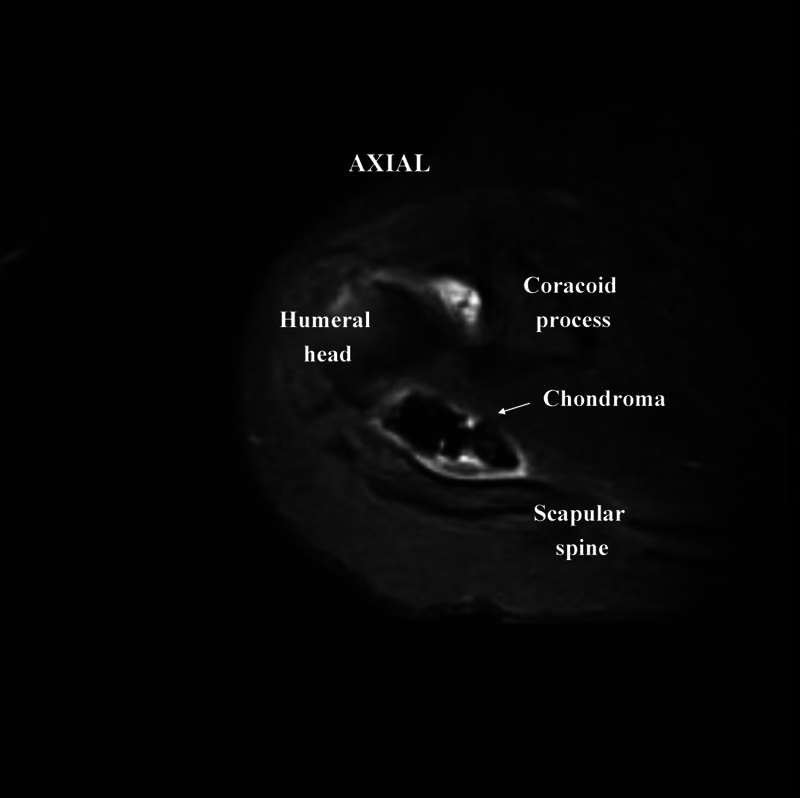
Magnetic resonance imaging of the shoulder (axial view, T2-weighted image)

Case 1: A 62-year-old female patient with a history of pain in her right shoulder after exertion. On examination, the shoulder presented mild edema and limited movement. The Neer impingement test and the Jobe and Speed tests were positive. Radiographs showed signs of impingement and calcifications. MRI revealed a rupture of the supraspinatus tendon and multiple calcifications in the bursa measuring 0.4 to 1.2 cm ( Figs. 1 and 2 ). The patient underwent video arthroscopic surgical treatment consisting of synovectomy, chondroma removal, and supraspinatus lesion repair. The anatomopathological examination confirmed synovial chondromatosis. The patient started physical therapy two weeks after surgery. During the outpatient follow-up, she presented improvement in pain, range of motion, and strength in her right shoulder.

**Fig. 3:**
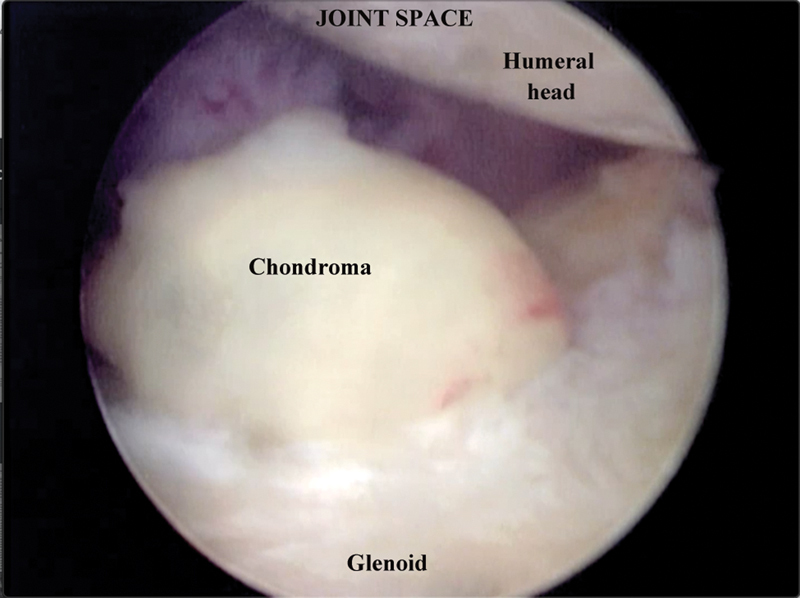
Intra-articular arthroscopic view.

**Fig. 4:**
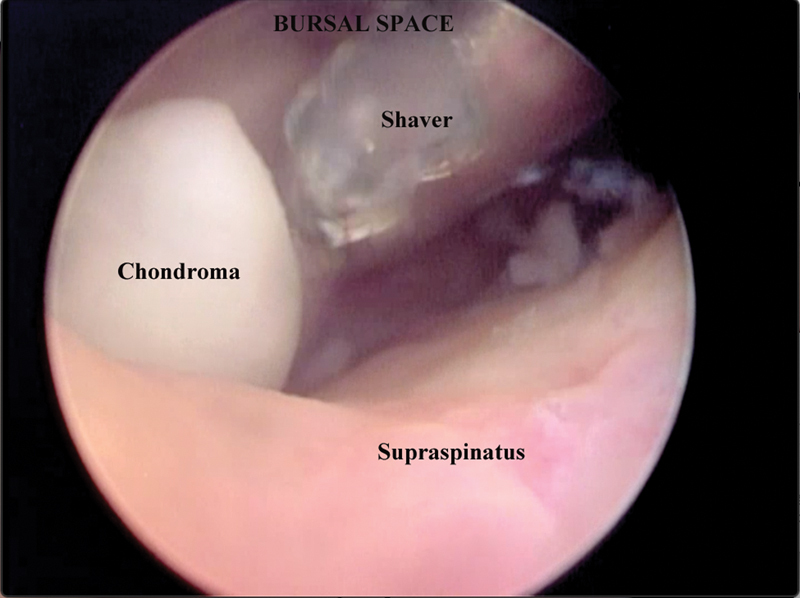
Subacromial arthroscopic view.

**Fig. 5:**
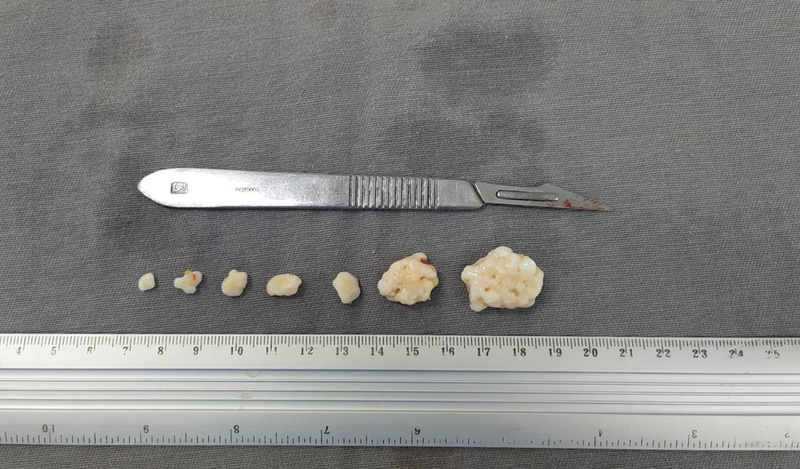
Macroscopic appearance of the removed chondromas.

Case 2: A 56-year-old female patient with a history of pain in her right shoulder. The physical examination revealed mild edema, limited movement, and a positive Jobe test. Radiographs showed signs of impingement and a hooked acromion with a spur. MRI revealed intense proliferative synovitis with small intra-articular nodules in the axillary recesses, bursitis, supraspinatus tendon rupture, extensive partial tear of the subscapularis tendon, and ruptured tendon of the long head of the biceps. The patient underwent video arthroscopic treatment for supraspinatus tendon injury repair, synovectomy, subacromial decompression, and removal of multiple loose bodies ( Figs. 3 , 4 , and 5 ). The anatomopathological examination confirmed synovial chondromatosis. The patient began physical therapy rehabilitation one month after surgery. Pain improved immediately after surgery, and shoulder function improved after six months of rehabilitation.

## Discussion

Shoulder CS is a rare report in the literature (<5%) due to its atypical location. 3 However, SC is a condition with well-defined characteristics. The classification into primary and secondary forms considers cartilaginous body number, shape, and size or the presence of a pre-existing disease. In shoulders, the secondary form is the most common, incidentally diagnosed in tests for a previous underlying disease. 5 Although CS is more common in men, we reported two female patients. The monoarticular characteristic is consistent with the literature. In both cases, CS had an association with rotator cuff syndrome.

The cartilaginous bodies originating from synovial metaplasia 3 may increase in size and undergo calcification 3 4 or endochondral ossification, causing joint erosions, pain, stiffness, and movement restriction .3 Therefore, imaging tests are essential, and their findings depend on the stage of the disease. Our patients underwent shoulder radiography and MRI, which detected loose bodies. In both cases, the MRI showed rupture of the supraspinatus tendon. Rotator cuff injury may result from the persistent presence of loose bodies in the subacromial region and impingement. 6

Although there is controversy about the best therapy for SC 2 7 8 9 and reports of spontaneous remission, 7 most of the literature supports surgical treatment. 1 2 9 Most cases under conservative therapies remain symptomatic or present with symptoms worsening before surgery. 9 Everything indicates that arthroscopic treatment is the gold standard, 9 but the need for synovectomy is not yet well established. 2 8 9 Disease recurrence has been reported since SC can affect the tendon sheath of the long head of the biceps and escape detection or due to its incomplete treatment with an isolated arthroscopic technique by not using arthrotomy or mini-open techniques when necessary. 9 Several authors described loose bodies removal with synovectomy. 8 However, Jeffreys (1967) concluded that only removing loose bodies was successful. 10 Milgram (1977) apud Maurice et al. 8 (1988) recommended synovectomy with free body removal for the initial SC stage and the isolated removal of these bodies in the late stage.

Some authors, such as Ramos et al. 7 (1997), prefer the simple removal of articular free bodies. However, when these bodies are close to the synovium, as in our cases, we propose the addition of arthroscopic synovectomy to increase procedural precision. Arthroscopy involves small incisions, allows assessing the entire glenohumeral joint, and facilitates rapid rehabilitation. 1 2 We believe that synovectomy and removing all loose bodies is the best therapy for shoulder CS, and resection of the bursa with the nodules in the subacromial region can minimize the occurrence of future rotator cuff injuries. 6 There are reports of recurrence when the synovial membrane is not excised, 3 favoring malignant transformation to synovial chondrosarcoma, although this event is rare. 1 3 The association of radiotherapy with treatment is questioned because there is little benefit in its use since metastasis developed by patients with previous CS is rare. 1

Rehabilitation with physical therapy is critical for shoulder function recovery and provides the good outcomes described in the literature. 9 In both cases, there was an improvement in pain and glenohumeral joint mobility. Long-term outpatient monitoring of these subjects is valuable since we must not neglect a potential recurrence. We should consider evaluation with imaging tests every 2 or 3 years for SC treatment. 2 5 8
