# Supplementary material for: Synovial Chondromatosis of the Shoulder: Report of Two Cases
Source: Rev Bras Ortop (Sao Paulo). 2024 Dec 27;59(Suppl 2):e180–3. doi: 10.1055/s-0044-1790596 (PMC11679617; doi:10.1055/s-0044-1790596)
Supplement: Related texts

# Research article: Condromatose sinovial do ombro: Relato de dois casos

**Keywords:** artroscopia, condromatose sinovial, membrana sinovial, ombro

## Abstract

A condromatose sinovial é uma afecção benigna incomum de caráter progressivo que favorece a metaplasia sinovial com a produção de tecido cartilaginoso em forma de corpos livres, encontrados nas articulações. Em casos raros, pode afetar o interior da articulação do ombro e apresentar-se com dor, edema e prejuízo da mobilidade. O diagnóstico é desafiador, requerendo técnicas de imagem e muitas vezes confirmado apenas após o tratamento cirúrgico e exame anatomopatológico. Os autores relatam dois casos de pacientes com quadros clínicos semelhantes de dor, edema e perda de mobilidade na articulação do ombro. A investigação contou com exames de imagem como radiografias e ressonância magnética, além do exame anatomopatológico, que confirmou a hipótese diagnóstica. O tratamento cirúrgico artroscópico com sinovectomia e remoção dos corpos livres, seguido de reabilitação fisioterapêutica, resultou na melhora clínica de ambas as pacientes. Com esse relato, os autores ressaltam a importância da investigação na suspeita de condromatose sinovial, devido ao seu quadro clínico inespecífico. Comparando os resultados obtidos com os propostos na literatura, concluímos que o tratamento cirúrgico com sinovectomia e remoção de corpos livres, associado a fisioterapia, é eficaz, e que o seguimento ambulatorial a longo prazo é necessário para detecção de recidiva.

## Introdução

A condromatose sinovial (CS) é uma artropatia excepcional, geralmente monoarticular, caracterizada pela proliferação e metaplasia do tecido sinovial, com a formação de corpos livres cartilaginosos na bainha dos tendões ou nos espaços articulares. 1 2 3 O padrão de acometimento inclui articulações diartrodiais, com destaque para o joelho, o quadril e o cotovelo, sendo escassa na literatura a descrição do envolvimento do ombro. 2 A etiologia ainda é desconhecida e a maior incidência da CS encontra-se na terceira até a quinta década de vida, sendo três vezes mais comum em homens. 3 O quadro clínico consiste em dor, crepitação, edema e limitação do movimento articular, 2 muitas vezes sem causa aparente. 1 A transformação maligna é incomum e não há relação direta com traumatismos ou processos inflamatórios. 4 O diagnóstico clínico é difícil, por não haver história e exame físico específicos. Assim, os métodos de imagem como radiografias, tomografia computadorizada e ressonância magnética tornam-se essenciais para identificar os diversos tipos de lesão e as fases da doença. 5 A confirmação do diagnóstico ocorre após o exame histológico do tecido sinovial e o tratamento de escolha para os pacientes sintomáticos é o cirúrgico. 1 3

## Relato de Caso

Caso 1: paciente do sexo feminino, 62 anos, com história de dor no ombro direito após esforço. Ao exame, o ombro apresentava leve edema e limitação dos movimentos. O teste do impacto de Neer e os testes de Jobe e Speed eram positivos. As radiografias demonstravam sinais de impacto e calcificações. O exame de ressonância magnética apresentou ruptura do tendão supra-espinal e múltiplas calcificações na bolsa, medindo de 0,4 a 1,2 cm ( Figs. 1 e 2 ). A paciente foi submetida a tratamento cirúrgico videoartroscópico, com sinovectomia e retirada dos condromas, além de reparo da lesão do supra-espinal. O exame anatomopatológico confirmou a condromatose sinovial. A paciente iniciou fisioterapia após duas semanas de pós-operatório. No acompanhamento ambulatorial, evoluiu com melhora da dor, da amplitude do movimento e da força do ombro direito.

**Fig. 1:** Imagem de ressonância magnética do ombro (corte coronal ponderado em T2).

**Fig. 2:** Imagem de ressonância magnética do ombro (corte axial ponderado em T2).

Caso 2: paciente do sexo feminino, 56 anos, com história de dor no ombro direito. Ao exame físico, apresentava leve edema, limitação dos movimentos e teste de Jobe positivo. As radiografias demonstravam sinais de impacto e acrômio ganchoso, associado a esporão. O exame de ressonância magnética apresentava intensa sinovite proliferativa com pequenos nódulos intra-articulares nos recessos axilares, bursite, ruptura do tendão supra-espinal, lesão parcial extensa do tendão subescapular e tendão da cabeça longa do bíceps roto. A paciente foi submetida a tratamento videartroscópico de reparo da lesão do tendão supra-espinal, sinovectomia, descompressão subacromial e retirada de múltiplos corpos livres ( Figs. 3 , 4 , e 5 ). O exame anatomopatológico confirmou a condromatose sinovial. A paciente iniciou a reabilitação fisioterapêutica com 1 mês de pós-operatório. Houve melhora da dor logo após o tratamento cirúrgico e da função do ombro com 6 meses de reabilitação.

**Fig. 3:** Visão artroscópica intra-articular.

**Fig. 4:** Visão artroscópica subacromial.

**Fig. 5:** Aspecto macroscópico dos condromas removidos.

## Discussão

A CS do ombro é um relato raro na literatura (<5%), devido à sua localização atípica, 3 apesar de ser uma patologia com características bem definidas. A classificação em formas primária e secundária considera o número, o formato e o tamanho dos corpos cartilaginosos ou a presença de doença pré-existente. No ombro, a forma secundária é a mais comum, incidentalmente diagnosticada em exames para uma doença de base prévia. 5 Apesar da CS ser mais comum em homens, em nosso relato os pacientes são do sexo feminino. A característica monoarticular é congruente com a literatura. Em ambos os casos, a CS apresentou relação com a síndrome do manguito rotador.

Os corpos cartilaginosos provenientes da metaplasia sinovial 3 podem aumentar de tamanho, sofrer calcificação 3 4 ou ossificação endocondral, provocando erosões articulares, dor, rigidez e restrição do movimento. 3 Por isso, exames de imagem são essenciais e seus achados dependem da fase da doença. Nossas pacientes foram submetidas a exames de radiografia e ressonância magnética do ombro, havendo a detecção de corpos livres. Nos dois casos, a ressonância magnética mostrou ruptura do tendão supra-espinal. A lesão do manguito rotador pode ser devida à presença persistente de corpos livres na região subacromial e ao impacto. 6

Apesar de haver controvérsias sobre a melhor terapia na CS, 2 7 8 9 além de relatos de remissão espontânea, 7 o tratamento cirúrgico é o mais respaldado pela literatura. 1 2 9 A maioria dos casos que utilizam terapias conservadoras permanecem sintomáticos ou ocorre o agravamento dos sintomas antes da cirurgia. 9 Tudo indica que o tratamento artroscópico é o *gold standard* 9 e a necessidade de realização da sinovectomia ainda não está bem estabelecida. 2 8 9 A recorrência da doença tem sido reportada devido ao fato de poder acometer a bainha do tendão da cabeça longa do bíceps e não ser detectada, ou ao seu tratamento incompleto quando da utilização de técnica artroscópica isolada, sem a realização de artrotomia ou técnicas *mini-* open, quando necessário. 9 A remoção dos corpos livres com sinovectomia é descrita por vários autores. 8 Entretanto, Jeffreys (1967) concluiu em seu estudo que apenas a remoção dos corpos livres foi muito bem sucedida. 10 Milgram (1977) apud Maurice et al. 8 (1988), recomendou a sinovectomia associada à remoção de corpos livres para a fase inicial e a remoção isolada daqueles na fase tardia.

Alguns autores, como Ramos et al. 7 (1997), preferem a simples retirada dos corpos livres articulares, mas quando há intimidade com a sinovial, como em nossos casos, preferimos acrescentar a sinovectomia artroscópica para melhor precisão do procedimento. A artroscopia possui incisões pequenas que permitem avaliar toda a articulação glenoumeral, além de facilitar a rápida reabilitação. 1 2 Acreditamos que a sinovectomia associada à remoção de todos os corpos livres é a melhor terapia para casos de CS do ombro, e a ressecção da bolsa junto aos nódulos na região subacromial pode minimizar a ocorrência de futuras lesões do manguito rotador. 6 Além disso, há registro de recidiva quando a membrana sinovial não é excisada, 3 favorecendo a transformação maligna para condrossarcoma sinovial, ainda que seja um evento raro. 1 3 A associação de radioterapia no tratamento é questionada devido ao fato de haver pouco benefício na sua utilização, já que metástase desenvolvida por paciente com CS prévia é um evento raro. 1

A reabilitação com fisioterapia é fundamental para a recuperação da função do ombro e para prover os bons resultados como descrito na literatura. 9 Em ambos os casos, obteve-se uma melhora não apenas da dor, como da mobilidade da articulação glenoumeral. O acompanhamento ambulatorial de longo prazo desses pacientes é importante, pois a possibilidade de recorrência não deve ser negligenciada. A avaliação a cada 2 ou 3 anos, com exames de imagem, deve ser considerado no tratamento desta patologia. 2 5 8
